# Identification of GBV-D, a Novel GB-like Flavivirus from Old World Frugivorous Bats (*Pteropus giganteus*) in Bangladesh

**DOI:** 10.1371/journal.ppat.1000972

**Published:** 2010-07-01

**Authors:** Jonathan H. Epstein, Phenix-Lan Quan, Thomas Briese, Craig Street, Omar Jabado, Sean Conlan, Shahneaz Ali Khan, Dawn Verdugo, M. Jahangir Hossain, Stephen K. Hutchison, Michael Egholm, Stephen P. Luby, Peter Daszak, W. Ian Lipkin

**Affiliations:** 1 Conservation Medicine Program, Wildlife Trust, New York, New York, United States of America; 2 Center for Infection and Immunity, Mailman School of Public Health, Columbia University, New York, New York, United States of America; 3 Chittagong Veterinary & Animal Sciences University, Chittagong, Bangladesh; 4 Programme on Infectious Disease and Vaccine Sciences, International Centre for Diarrheal Disease Research, Bangladesh (ICDDR,B), Dhaka, Bangladesh; 5 454 Life Sciences, Branford, Connecticut, United States of America; Cornell University, United States of America

## Abstract

Bats are reservoirs for a wide range of zoonotic agents including lyssa-, henipah-, SARS-like corona-, Marburg-, Ebola-, and astroviruses. In an effort to survey for the presence of other infectious agents, known and unknown, we screened sera from 16 *Pteropus giganteus* bats from Faridpur, Bangladesh, using high-throughput pyrosequencing. Sequence analyses indicated the presence of a previously undescribed virus that has approximately 50% identity at the amino acid level to GB virus A and C (GBV-A and -C). Viral nucleic acid was present in 5 of 98 sera (5%) from a single colony of free-ranging bats. Infection was not associated with evidence of hepatitis or hepatic dysfunction. Phylogenetic analysis indicates that this first GBV-like flavivirus reported in bats constitutes a distinct species within the *Flaviviridae* family and is ancestral to the GBV-A and -C virus clades.

## Author Summary

Bats are important reservoirs for emerging zoonotic viruses with significant impact on human health including lyssaviruses, filoviruses, henipaviruses and coronaviruses. Opportunities for transmission to humans are particularly prominent in countries like Bangladesh, where people live in close association with bats. Whereas previous studies of bats have employed assays that test for known pathogens, we present the first application of an unbiased molecular approach to pathogen discovery in this reservoir for emerging zoonotic disease. Unbiased pyrosequencing of serum from *Pteropus giganteus* bats enabled identification of a novel flavivirus related to Hepatitis C and GB viruses. Viral nucleic acid was present in 5 of 98 (5%) sera, and in the saliva of one animal. Sequence identification of two strains of the virus, tentatively named GBV-D, suggests *P. giganteus* as a natural reservoir. Detection of viral nucleic acid in saliva provides a plausible route for zoonotic transmission. Phylogenetic analysis indicates that GBV-D is ancestral to GBV-A and -C, and separate from the recently classified genus *Hepacivirus*. Our findings provide new insight into the range of known hosts for GB-like viruses and demonstrate the power of unbiased sequencing to characterize the diversity of potentially zoonotic pathogens carried by bats and other reservoirs.

## Introduction

Bats (order *Chiroptera*), after rodents, comprise the most diverse group of mammals with more than 1,100 species. They are present on six continents, often have substantial habitat overlap with humans [1] and harbor several zoonotic viruses causing significant human morbidity and mortality, including Ebola- and Marburgvirus, Nipah virus (NiV), and SARS-like coronaviruses [2]–[5]. Proximity of bats to human populations may facilitate the zoonotic transmission of viruses either through direct contact, via amplifying domestic animal hosts, or through food-borne routes [6]–[8].

The current study was set up as part of a viral discovery effort to target key wildlife reservoirs in emerging disease hotspots. Bangladesh is a ‘hotspot’ for emerging zoonotic diseases [9], with a relatively high diversity of wildlife that likely harbors new zoonotic pathogens, one of the densest human populations on the planet, and a high level of connectivity between people, domestic animals and wildlife. In Bangladesh and India, frugivorous *Pteropus giganteus* bats have been identified as a reservoir for NiV [10], [11], which has been recognized as the cause of several outbreaks of encephalitis [12]–[14]. *Pteropus giganteus* bats are common throughout the Indian subcontinent, living in close association with humans and feeding on cultivated fruit [14]. NiV transmission from bats to humans has been linked with the harvest and consumption of raw date palm sap, which becomes contaminated with bat feces, urine or saliva overnight when bats such as *P. giganteus* come to feed from the collecting pots [14], [15]. Date palm sap or other foods eaten by both bats and people, may also serve as a vehicle for transmission of other bat-borne agents.

Several zoonotic flaviviruses, including Japanese encephalitis virus, West Nile virus, and Kyasanur forest virus have been identified in bats; however, to date, GB viruses have not [1]. GB viruses A and C (GBV-A and -C) represent two recently identified species that are currently unassigned members of the family *Flaviviridae* [16]. GBV-A viruses have been described in New World primates and are not known to infect humans [17]–[19], while GBV-C (also known as Hepatitis G virus (HGV)) have frequently been isolated from humans in many regions of the World, including India and Bangladesh [19]–[23], and from wild chimpanzees (*Pan troglodytes*) in Africa [24], [25]. Here we describe discovery of a virus in the serum of healthy bats in Bangladesh, tentatively named GB virus D (GBV-D), that is distantly related to GBV-A and -C and represents a new member of the family *Flaviviridae*.

## Materials and Methods

### Ethics statement

Every effort was made to minimize bat stress and avoid injury during capture, restraint, and sampling procedures. This study was conducted following Wildlife Trust institutional guidelines under IACUC approval G2907 issued by Tufts New England Medical Center, Boston, Massachusetts.

### Bat sample collection

**Figure 1:**
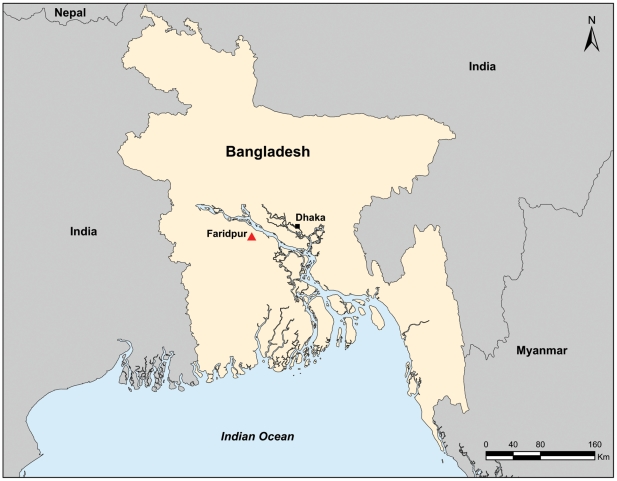
Map showing the location of the bat colony in Faridpur district, Bangladesh from which GBV-D was identified.

As part of a longitudinal surveillance study of Nipah virus in bats, 98 free-ranging *P. giganteus* bats were caught from a colony of approximately 1800 individuals in the Faridpur district of Bangladesh in December 2007 (**Figure 1**). Each bat was anesthetized using isoflurane gas; morphometric measurements (weight, forearm length, head length, and body condition) were taken and bats were aged [10]. Each bat was marked for future identification using an RFID microchip (AVID corp, www.avidid.com) implanted subcutaneously between the scapulae. Three mL of blood were collected and placed into serum separator tubes (vacutainer; Becton Dickinson, Franklin Lakes, NJ, USA). Serum was allowed to separate overnight at 4°C then drawn off without centrifugation and immediately frozen using a liquid nitrogen dry shipper. To inactivate potentially infectious agents, serum samples were heat-treated at 56°C for 30 min and then stored at −70°C. For RNA extraction, 250 µL of serum was added to 750 µL Tri-Reagent LS (Molecular Research Center, Cincinnati, OH, USA). Saliva was collected from the bat's throat using a sterile cotton swab. Urine was collected either by catching urine in a 1.0 mL sterile cryovial while the bat was urinating, or by urethral swab. Urine and saliva swabs were immediately placed into 1 mL Tri-Reagent LS and frozen in liquid nitrogen.

### Unbiased high-throughput pyrosequencing (UHTS)

Total RNA from serum was extracted for UHTS analysis to screen for the presence of microorganisms. Five microliters of total RNA from each bat were combined into 4 pools: 4 pregnant bats; 4 non-pregnant female bats, and 2 pools of 4 adult male bats, respectively. Reverse transcription (RT) was performed on DNase I-treated (DNA-free, Ambion Inc., Austin, TX, USA) RNA pools to generate cDNA using Superscript II RT (Invitrogen, Carlsbad, CA, USA) and random octamers linked to a defined arbitrary, 17-mer primer sequence tail (MWG, Huntsville, AL, USA) [26]. After RNase H treatment cDNA was amplified by the polymerase chain reaction (PCR), applying a 9∶1 mixture of the defined 17-mer primer sequence and the random octamer-linked 17-mer primer sequence, respectively [27]. Products of >70 base pairs (bp) were selected by column purification (MinElute, Qiagen, Hilden, Germany) and ligated to specific linkers for sequencing on the 454 Genome Sequencer FLX (454 Life Sciences, Branford, CT, USA) without DNA fragmentation [28], [29]. Sequences were analyzed using software applications implemented at the GreenePortal website (http://tako.cpmc.columbia.edu/Tools/).

### Genome sequencing

Multiple forward and reverse primers for RT-PCR (available upon request) were designed using the sequences obtained by UHTS in order to fill gaps between fragments. Amplifications were performed with Bio-X-act (Bioline, London, UK) according to manufacturer's protocols. Products were size fractionated by electrophoresis and directly sequenced in both directions with ABI PRISM Big Dye Terminator 1.1 Cycle Sequencing kits (Perkin-Elmer Applied Biosystems, Foster City, CA, USA) at a commercial facility (Genewiz, South Plainfield, NJ, USA). Additional methods applied to obtain the genome sequence included touch-down PCR [30], 2-step walking PCR [31], and 3′- and 5′- RACE (Invitrogen).

### Quantitative real-time PCR

A real time Taqman PCR assay was developed to screen bat samples for GBV-D. Reactions were performed in a 25 µL volume by using commercial Taqman Universal Master Mix (Applied Biosystems, Foster City, CA, USA). Primers and probe were designed to target a 60 nt region in the NS4A gene region: Fadi-forward, 5′- gCAgCTgCgTgTgCCA; Fadi-reverse, 5′- ACACCCATgATgTTACCACgAC; Fadi-probe, 5′- FAM- AggACCCggTCgCTCCAgCA-T-BQX (TIB Molbiol, Adelphia, NJ, USA). Cycling conditions were: 50°C for 2 min, and 95°C for 10 min, followed by 45 cycles at 95°C for 15 sec and 60°C for 1 min. Thermal cycling was performed in an ABI 7300 real-time PCR system (Applied Biosystems).

### Serum chemistry

A liver function panel was conducted at the International Center for Diarrheal Disease Research (Dhaka, Bangaldesh) using non heat-treated bat sera (Automated Chemistry Analyzer AU 640, Olympus Corporation, Tokyo, Japan). The following parameters were analyzed: total protein, albumin, globulin, albumin∶globulin ratio, total cholesterol, total bilirubin , alkaline phosphatase, alanine transferase, aspartate aminotransferase, gamma glutamyltransferase , and lactate dehydrogenase.

### Phylogenetic and sequence analyses

Sequence alignments were generated with ClustalW software [32] and phylogenetic relationships deduced using Geneious software [33]. Statistical significance was assessed by bootstrap re-sampling of 1000 pseudoreplicate data sets. Sequence relations were determined from p-distance matrices calculated with pairwise deletion for missing data and homogeneous patterns among lineages based on ClustalW alignments as implemented in MEGA software [34]. Sliding window similarity analysis was performed using SimPlot [35]. Potential signalase cleavage sites, glycosylation sites, and phosphorylation sites were analyzed using the respective prediction servers available at the Center for Biological Sequence Analysis (http://www.cbs.dtu.dk/services/).

## Results

### Identification of a GB-like agent from bats

Total RNA from the serum of healthy bats captured at a roost in the Faridpur district of Bangladesh was extracted for UHTS analysis. Extracts of 16 individual bats were combined into 4 pools consisting of 4 pregnant adult bats, 4 non-pregnant adult female bats, or 2×4 adult male bats. Each pool yielded between 1,400 and 2,000 assembled contigs or singlton reads (representing 50,000–75,000 reads ranging in size from 31–328 nt). Two reads of 238 and 215 nucleotides (nt) derived from the pregnant bat pool had distant homology to GBV-A sequences at the deduced amino acid (aa) level in the E2 and NS4A gene regions respectively (BLASTX); no homology was detected by searches at the nt level (BLASTN; local copy of the executables with standard settings except that the reward for a nucleotide match was set to 2 instead of 1). No viral sequences were detected in other pools at the nt or aa levels. Screening of the individual RNA preparations from the pregnant bat pool using primers derived from the UHTS reads confirmed the presence of the GBV-like sequence in the serum of bat 93. A quantitative real time PCR assay indicated a load of approximately 30 000 RNA copies in bat-93 serum extract, and identified an additional 4 positive bat sera from the original 98 samples (5/98; 5%), indicating serum loads ranging from 350 to 70,000 RNA copies per assay. These positive samples came from male bats that were not included in the initial UHTS pools. Extracts of saliva from the five positive bats indicated a load of approximately 200 RNA copies in bat 93; no signal was obtained with urine extracts from the five positive bats.

### Genomic characterization of GBV-D

**Figure 2:**
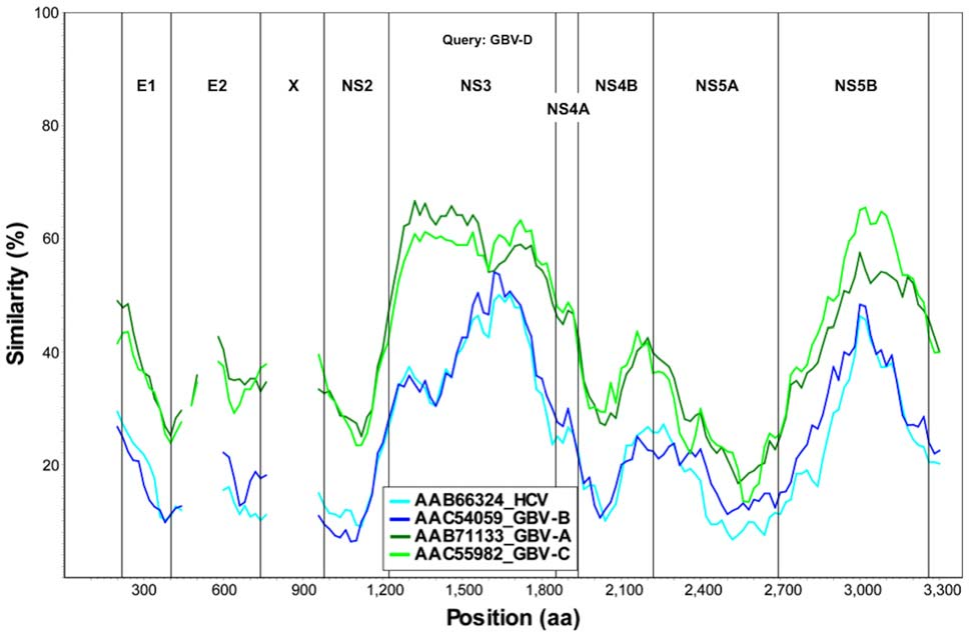
Sliding window similarity analysis between GBV-D and other GBV and hepaciviruses (amino acid sequence; window, 160; step, 20).

**Figure 3:**
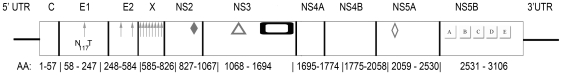
Genomic organization of GBV-D, a novel flavivirus identified in the sera of frugivorous bats in Bangladesh. Arrows, glycosylation sites; solid diamond, active center sites H_921_, E_1011_, and C_1032_ in the autocatalytic NS2/NS3 endoprotease domain; triangle, catalytic triad H_1123_, D_1147_, S_1204_ of NS3 serine protease; rectangle, NS3 helicase and DEAD-like helicase motifs; open diamond, zinc finger motif; and NS5 polymarase motifs A (T_2744_VDAICFDSCIT), B (R_2802_ASGVLTTSSSNCISSFLKVSAAC), C (F_2835_LIHGDDVMII), D (L_2876_DTAQSCSA),and E (H_2900_YFLSTDFR) motifs.

Near full-length genome sequence was generated from bat-93 and a second positive serum (bat 68), applying primers crossing gaps between UHTS reads as well as touch-down PCR [30], 2-step walking PCR [31], and 3′- and 5′-RACE (Invitrogen) protocols. The two genome sequences were 96% identical at the nt level (GenBank Accession nos. GU566734 and GU566735), indicating two strains of the same virus. Comparison of deduced polyprotein sequence to other GBV and hepaciviruses indicated highest nt and aa sequence identities to GBV-A and -C (**Table 1**, **Figure 2**). The genomic sequence of the GBV-like virus identified in *P. giganteus* bats, tentatively named GBV-D, comprises 9,633 nt with 52 nt of potentially 5′-untranslated region (UTR), one continuous open reading frame (ORF) of 9318 nt (3106 aa) and 265 nt of 3′-UTR (**Figure 3**).

**Table 1:** Percent sequence similarity between GBV-D (bat-68), -A, -C, and hepaciviruses.

nt* aa	GBV-D	GBV-C	GBV-A	GBV-B	HCV-1
GBV-D		48	46	39	41
GBV-C	41		55	41	44
GBV-A	39	47		37	38
GBV-B	25	24	24		41
HVC-1	22	22	22	27	

*Sequence similarity at nt level in upper right, and at aa level in lower left portion of the table.

Mature structural proteins in GB viruses, as well as other flaviviruses, are the product of cleavage by host signal peptidase [36]. In GBV-D the first potential signal sequence cleavage site is present after a stretch of 57, largely basic aa (6 kDa, pI = 12), followed by sequence homologous to E1 (pfam 01539, http://pfam.sanger.ac.uk/) (**Figure 3**). The single glycosylation site N_177_IT present in that sequence is located in a position comparable to GBV-C, -A, -B and HCV glycosylation sites. Identification of the downstream E2 termini is less apparent as the next 580 aa contain multiple potential signal sequences and 10 potential glycosylation sites that indicate no homology to hepaciviral E2/NS1 (pfam 01560), until the sequence aligns with N-terminal NS2 motifs (pfam 01538) (**Figure 2** **,** **Figure 3**). However, despite similarity to pfam 01538 no signal sequence compatible with cleavage at A_759_/A was found; cleavage may occur at G_826_/R, which combined with potential signalase cleavage at A_584_/F may indicate the existence of a heavily glycosylated potential 26 kDa product instead of the p7 trans-membrane protein identified in HCV [37]–[39] or the 13 kDa variant described in GBV-B [40], [41]. Conserved C-terminal motifs of the autocatalytic NS2/NS3 endoprotease domain are compatible with NS2/NS3 cleavage at S_1067_/A and comparable to other GBV and HCV [42]. **Figure 3** indicates potential cleavage sites for NS3 (peptidase S29, pfam 02907; DEAD box helicase, pfam 07652; helicase C, pfam 00271), NS4A (pfam 01006), NS4B (pfam 01001), NS5A (domain-1a zinc finger, pfam 08300; domain-1b, pfam 08301), and NS5B (pfam 00998).

Conserved aa motifs were recognized in NS proteins. RNA-dependent RNA polymerase (RdRp) motifs in RdRp block III that are conserved with respect to other GBV and hepaciviruses were identified in NS5B (**Figure 3**) [43]–[46]. Potential phosphorylation sites are present at multiple serine (9), threonine (14) and tyrosine (4) residues in NS5A, compatible with its possible function as a phosphorylation-regulated mediator of viral replication [47]. However, significant conservation of primary sequence is not obvious for phosphorylation sites, proline-rich, or interferon-sensitivity determining region motifs [48]–[50]. The C-terminal portion of NS3 has homology to conserved NTPase/helicase motifs [51]; the N-terminal portion includes conserved active triad residues H_1123_, D_1147_, S_1204_ of serine protease [52], the viral protease responsible for cleavage of mature non-structural proteins [53]. Likewise, the active triad H_991_, E_1011_, C_1032_ of the *cis*-acting protease activity in the C-terminal portion of NS2 is conserved with respect to other GBV and HCV [42]. The only other discernable motif identified was a well-conserved N_75_ C/D C motif at the N-terminus of E1 (**Figure 3**) [54].

### Phylogenetic analysis

**Figure 4:**
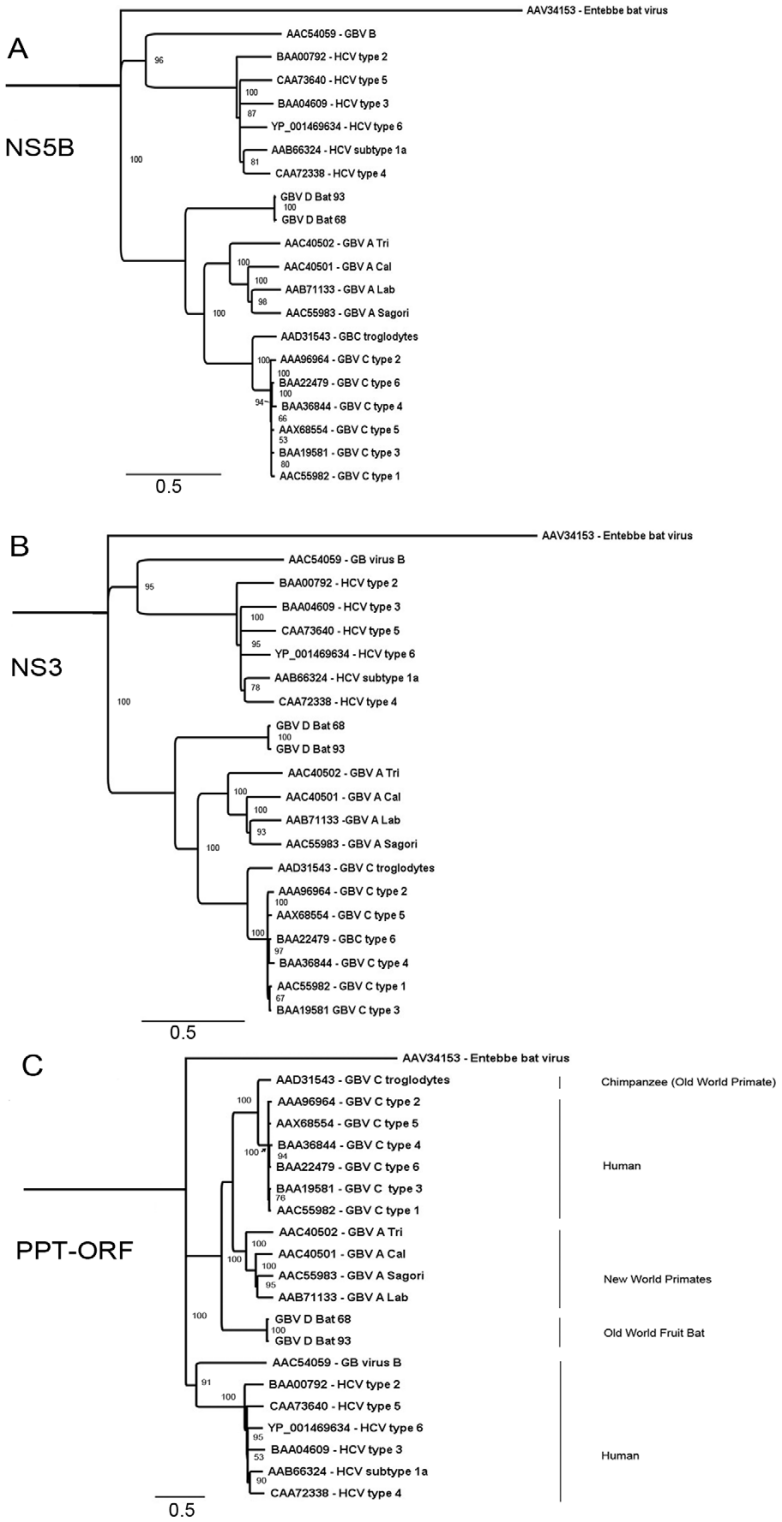
Phylogenetic relationship of GBV-D to other GBV and hepaciviruses. GBV-D amino acid sequences for A: NS5B, B: NS3, and C: the polyprotein (PPT) were analyzed in comparison to representative sequences of GBV-A, -B, -C and hepatitis C viruses. GenBank accession numbers for the respective sequences are indicated. Entebbe bat virus was used as an outgroup; distance in substitutions per site is indicated by scale bars; percent bootstrap support for values greater than 85% is indicated at respective nodes.

Phylogenetic analysis of GBV-D was performed in comparison to selected representatives of GBV-A, GBV-B, GBV-C and HCV. Analysis of NS5B aa sequence (**Figure 4A**) confirmed a closer relationship of GBV-D to GBV-A and -C than to GBV-B or HCV as also indicated by pairwise sequence comparisons (**Table 1**). The same relationships were also apparent when NS3, or the complete polyprotein sequence were analyzed (**Figure 4B and C**, respectively). All three trees show GBV-D consistently at the root of the GBV-A/-C viruses, indicating an independent phylogenetic clade compatible with a separate species distinct from the recently created genus *Hepacivirus* [16].

### Serum chemistries

A liver serum chemistry panel was conducted on sera from 15 bats, the five GBV-D infected and 10 non-infected animals. Standard assays to detect hepatitis and/or impaired liver function were performed [55]. Levels of total protein, alanine transferase, aspartate aminotransferase and total cholesterol were within published ranges reported for *P. giganteus*, except for bat 33 (infected) and bat 73 (uninfected), which had modest elevation in aspartate aminotransferase. Reference values for albumin, globulin, albumin∶globulin ratio, total bilirubin, alkaline phosphatase, gamma glutamyltransferase and lactate dehydrogenase are not available for *P. giganteus*, however, values were comparable to those reported for other *Pteropus* species [56]. Mean values did not significantly differ between infected and uninfected bats (**Table 2**).

**Table 2:** Liver function values from *Pteropus giganteus* bats.

	T.Prot (g/dL)	Alb (g/dL)	Glob	A∶G	T. Chol (mg/dL)	T. Bili (mg/dL)	Alk Phos	ALT (U/L)	AST (U/L)	GGT (U/L)	LDH (mg/dL)
Ref Range (human)	64.0–82.0	34–50	23–35	1.1–1.8	3.6–6.3	.01–19	30–120	.01–41	.01–38	9–40	135–225
Ref Range (*P. giganteus*)	65–84	30–41			0–2.3			1.2–84.6	30.2–141.0		
Bat ID											
1	86	50.7	35.3	1.44	1.65	1.8	1572..4	13.9	67.6	87.90	53.4
3	79.7	49.4	30.3	1.63	2.74	2.1	605.7	20.2	67.4	49.20	95
**4**	**70.4**	**42.9**	**27.5**	**1.56**	**0.5**	**2.3**	**532.1**	**12.3**	**66.1**	**75.40**	**76.7**
21	76	48	28	1.71	1.06	1.2	794.9	23.8	83.7	35.70	244.5
23	71.5	46.2	25.3	1.83	1.49	1.7	1650	12	115.1	102.00	96
**33**	**80.5**	**43.8**	**36.7**	**1.19**	**1.09**	**2.1**	**891.6**	**22.2**	**146**	**32.10**	**752.4**
36	89.9	51	38.9	1.31	1.22	2.5	580.4	11.1	109.4	69.00	114.4
**37**	**76.6**	**47**	**29.6**	**1.59**	**0.85**	**1.3**	**205.8**	**12.6**	**50.3**	**72.50**	**133.9**
39	70.4	43.3	27.1	1.6	0.5	1.8	328.7	20.5	77.8	31.00	82.6
**68**	**78.9**	**44.9**	**34**	**1.32**	**2.54**	**1.2**	**813.3**	**7.9**	**65.6**	**46.10**	**111.8**
73	82.4	46.1	36.3	1.27	1.17	1.8	958.8	19.7	185.4	37.00	859.4
82	72.3	43.8	28.5	1.54	0.5	1.4	458.4	22.1	82.1	48.50	350.3
84	82.6	46.2	36.4	1.27	1.43	2.7	923.6	22.9	107.6	46.40	176
**93**	**82.1**	**47.5**	**34.6**	**1.37**	**1.46**	**2.6**	**262**	**8.6**	**59.9**	**47.10**	**105.2**
96	76.5	46.8	29.7	1.58	0.5	1.1	574.6	20.9	79.4	101.80	203.7
Mean uninfected	78.73	47.15	31.58	1.518	1.226	1.81	763.9	18.71	97.55	60.85	227.53
**Mean infected**	**77.7**	**45.22**	**32.48**	**1.406**	**1.288**	**1.9**	**540.96**	**12.72**	**77.58**	**54.64**	**236**
STdv uninfected	6.52	2.63	4.71	0.19	0.68	0.52	391.61	4.62	35.31	27.40	239.64
**STdv infected**	**4.56**	**1.99**	**3.80**	**0.17**	**0.78**	**0.62**	**311.19**	**5.71**	**38.77**	**18.63**	**289.40**

No indication of hepatitis or impaired liver function was observed; no significant differences between mean values for infected (bold) or non-infected bats were apparent.

The following parameters were analyzed: Total Protein (T. Prot); Albumin (Alb), Globulin (Glob), Alb∶Glob ratio, Total Cholesterol (T. Chol); Total Bilirubin (T. Bili); Akaline Phosphatase (Alk. Phos); Alanine Transferase (ALT); Aspartate Aminotransferase (AST); Gamma Glutamyltransferase (GGT) and Lactate Dehydrogenase (LDH).

## Discussion

Molecular analyses of sera from *Pteropus giganteus* bats from Faridpur, Bangladesh led to the identification of a 9,633 nt sequence consistent in genomic organization with known GBV and other species within the family *Flaviviridae* [16]. Whereas previous studies of bats have employed assays that test for known pathogens, ours is the first report of an unbiased molecular approach to pathogen discovery in this important reservoir of emerging infectious diseases. The modest yield of novel microbial sequences may reflect the choice of sample (e.g., serum vs feces, tissue or another specimen), competition between host and microbial template during unbiased amplification, or both. Efforts to address template competition are under way that include subtraction of host nucleic acids or the use of semi-random primers that do not amplify host sequences. Such efforts will likely enhance the sensitivity and throughput of unbiased sequencing technologies for pathogen discovery.

The discovery of this chiropteran flavivirus broadens both the taxonomical and geographical distribution of GB-like viruses. Three types of GB viruses have been described: GBV-A, -B and -C [18], [19], [24], [25], [54], [57]. GBV-B, which has never been found in humans and was only reported in captive tamarins after serial passage of the original human GB serum [58], is most closely related to HCV and was recently classified together with HCV into a new genus, *Hepacivirus*, within the family *Flaviviridae* [16]. GBV-A and -C remain unclassified members of the family. GBV-A have been isolated from several New World monkeys. Different genotypes appear to be associated with specific monkey species of the genera *Saguinus*, *Callithrix* (*Callitrichidae* family) and *Aotus* (*Aotidae* family), without any clinical signs associated with infection [24], [54], [57]. GBV-C have been isolated from humans with non-A-E hepatitis; however, its pathogenicity is unknown and the virus is widespread in the human population [21], [59]–[61]. Population studies showed that GB viruses are enzoonotic and species-specific within both Old and New World nonhuman primates as well as humans, and have likely co-evolved with their hosts over long periods of time [62]. Previously, the only GBV found in the Old world was GBV-C from chimpanzees (in Africa) and humans. Although GBV-C were found in humans, GB viruses have not been previously reported in primates or other animals on the Indian subcontinent.

GBV-C and -A are remarkable for a truncated or missing capsid (C) protein [18], [19]. Due to exhaustion of our samples we were unable to complete assessment of the 5′-terminal sequence; nonetheless, RACE experiments suggest that GBV-D likely codes for a short basic peptide, instead of a full-length C protein. The first methionine (M_1_) predicts a peptide of 57 aa (pI = 12); however, the more favorable Kozak context [63] of M_3_ indicates a 55 aa peptide. After signalase cleavage from the polyprotein precursor, this peptide may be functional, possibly influencing maturation of, or directly binding to, the E1 and/or E2 glycoproteins.

Phylogenetic analyses of NS5B, NS3 and complete polyprotein sequence place GBV-D at the root of the GBV-A and -C clades and are consistent with a model wherein GBV-D is ancestral to GBV-A and -C clades. Mixed relationships indicative of recombination events [64] were not evident (**Figure 2**, **Figure 4**). Both pteropid bats and chimpanzees are restricted to the Old World. While the range of chimpanzees (Africa) and *P. giganteus* (the Indian subcontinent) do not overlap, it is possible that other primate species in Bangladesh or India, such as macaques, or other fruit bats in Africa such as *Eidelon spp.*, whose range overlaps that of chimpanzees, may carry related viruses. While GBV-A is only known from primates of the New World, an African origin has been suggested for GBV-C based on a 12-aa indel sequence in NS5A [65]. Although the NS5A sequence of GBV-D, similar to that of GBV-A, appears elongated in the indel region, compatible with their respective earlier phylogenetic branching compared to GBV-C, little sequence conservation is observed in that region.

The bats in this study, like primates infected with their associated GBV [66], all appeared to be healthy. The lack of chemical evidence of hepatic inflammation or dysfunction suggests that this virus may not target hepatic cells in bats. This is consistent with the behavior of GBV-A in its natural primate hosts [54]. In contrast, elevated alanine transferase levels and mild hepatitis are observed in experimental infections of macaques with GBV-C isolates from humans [67]. Five percent of the bats we studied were infected with one of at least two different strains of GBV-D, which suggests widespread viral circulation within this species. The observation that bats are asymptomatically infected with diverse strains that constitute a distinct phylogenetic clade is compatible with a co-evolutionary relationship between GBV and their hosts [57], [62], and supports the hypothesis that *P. giganteus* bats may be a natural reservoir for GBV-D. In one case we were able to detect GBV-D nucleic acid in saliva. This suggests a potential route for viral transmission via fighting or grooming behavior, or via food shared by bats.

*Pteropus giganteus* is a frugivorous bat species that carries NiV, a zoonotic paramyxovirus [10], [11]. This species lives in close association with humans in Bangladesh and bats have been observed drinking from (and urinating into) date palm sap collecting pots [14]. Human consumption of contaminated palm juice is proposed to be a major route of NiV transmission [68]. Although it is unclear whether infectious virus was present in bat saliva, the observation that saliva can contain GBV-D nucleic acids provides a biologically plausible mechanism for transmission from infected bats to other hosts. While it is currently unknown whether GBV-D virus occurs in humans, up to 20% of non-A-E hepatitis cases remain unexplained [19].
